# The association of multigenerational family characteristics with loneliness and social isolation in older adults

**DOI:** 10.1038/s41598-025-30227-7

**Published:** 2025-12-04

**Authors:** Zeinab Baheshmat, Afsaneh Bakhtiari, Hossein-Ali Nikbakht, Shabnam Omidvar

**Affiliations:** 1 Student Research Committee, Health Research Institute, Babol University of Medical Sciences, Babol, Iran; 2 Social Determinants of Health Research Center, Health Research Institute, Babol University of Medical Sciences, Babol, Iran

**Keywords:** Loneliness, Social isolation, Older adults, Health care, Medical research, Risk factors

## Abstract

Loneliness and social isolation are widespread among older adults, especially in aging societies undergoing demographic and social transitions. Despite growing global interest, few studies have examined how multigenerational family characteristics contribute to these outcomes in later life. This study explored the associations between loneliness and social isolation in older adults and the demographic, health, and socioeconomic features of three family generations. This community-based cross-sectional study used a two-stage stratified random sampling method. Data were collected through structured interviews using standardized questionnaires. A total of 185 older adults (first generation), 185 adult children (second generation), and 185 grandchildren (third generation) participated. Standardized tools, including the De Jong Gierveld Loneliness Scale and the Social Isolation Questionnaire by Chalabi and Amirkafi, were used to assess outcomes. Sociodemographic and health characteristics were recorded across all generations. Statistical analyses included descriptive statistics, chi-square, t-tests, ANOVA, and both univariate and multivariate linear regression models. The prevalence of loneliness and social isolation in the older adults sample was 68.6% and 29%, respectively. Financial sufficiency in older adults was significantly associated with lower loneliness levels (B = − 0.69, *p* = 0.039), and rural residence predicted lower social isolation (B = − 4.43, *p* = 0.033). Among second-generation variables, only adult children’s residential proximity to the older parent significantly predicted lower social isolation (B = 3.73, *p* = 0.020). Characteristics of grandchildren showed no significant association with either outcome. Financial adequacy specifically reduces loneliness, while rural residence and children’s proximity protect against social isolation. Other intergenerational factors showed limited impact. Findings advocate for targeted interventions ensuring economic security and family proximity, offering more effective strategies than broad multigenerational approaches for promoting elder well-being.

**Supplementary Information:**

The online version contains supplementary material available at 10.1038/s41598-025-30227-7.

## Introduction

Loneliness and social isolation among older adults have become critical global public health concerns. The World Health Organization now recognizes them as risk factors with adverse effects comparable to smoking and physical inactivity, contributing to increased risks of depression, cardiovascular illness, cognitive impairment, and premature death^1–3^. While global research has explored various individual predictors such as health status or community involvement, less attention has been paid to the multigenerational family context, particularly in Middle Eastern societies where family remains a central unit of social support^3,4^.

In several Western and East Asian studies, close ties with children and grandchildren, through co-residence or frequent contact, have been shown to reduce loneliness and social withdrawal^5,6^. For instance, a national cohort study in China found that intergenerational proximity significantly buffered the effects of widowhood and chronic illness on elder isolation^7^. Similarly, a longitudinal analysis in Finland reported emotional transmission of loneliness from parents to children, highlighting the bidirectional nature of social vulnerability^8^. However, such findings are not always consistent across cultural settings. In Iran, while family traditionally provides strong emotional and instrumental support, recent urbanization, declining fertility, and migration patterns have weakened intergenerational proximity, especially in urban settings^9,10^. Although some Iranian studies have examined loneliness among older adults, most focus on psychological or socioeconomic factors^11^. A recent survey in Tehran, identified depression and low income as key predictors of loneliness but did not assess family dynamics^12^. Moreover, few studies in Iran have addressed how characteristics of adult children or grandchildren, such as proximity, health, and financial status, may relate to elders’ social well-being, despite growing policy interest in age-friendly community structures^13,14^. The theoretical basis of the present study draws on the convoy model of social relations and intergenerational solidarity theory, which both emphasize the role of family structure, functional support, and geographic closeness in shaping social connectedness and well-being in late life^15^.

The present study addresses this gap by employing a multigenerational framework to explore how a range of characteristics across three generations, elders, adult children, and grandchildren, are associated with perceived loneliness and social isolation among older adults in Iran. By integrating detailed demographic, health, and residential information for each generation, this study offers new insights into how family structure and proximity intersect with aging outcomes. It also responds to broader global calls for culturally contextualized research on the social determinants of health in aging societies^2,4^. In doing so, it contributes a nuanced, empirically grounded perspective to the literature on intergenerational influences on late-life social connectedness.

## Method

### Study design

This community-based cross-sectional study was conducted from September 2022 to April 2023 in Jouybar, Mazandaran Province, Iran, to explore the link between multigenerational analysis of family characteristics and social isolation and loneliness in older adults. The study protocol received ethical approval from the Institutional Review Board of Babol University of Medical Sciences (Ethics Code: IR.MUBABOL.REC.1401.073). All methods were performed in accordance with the relevant guidelines and regulations, including the ethical standards of the Declaration of Helsinki. Written informed consent was obtained from all participants after full explanation of study procedures.

### Participants

The study examined family networks across three generations. To ensure comparability, participation was limited to married older couples over 60 in Juybar city, their married adult children, and grandchildren. Families were eligible if the older couple had not remarried, maintained regular family contact, and showed functional independence on the ADL test. Exclusion criteria were severe sensory or language impairments, serious illness, or ongoing treatment for Alzheimer’s disease or depression; as such conditions could alter social behavior and confound interpretation.

In households with more than one adult child, one was randomly selected from the family roster. For the third generation, the grandchild of that selected child participated, maintaining lineage consistency within each family unit. This procedure supported comparability while keeping data collection feasible. Written informed consent was obtained from all participants; for minors, consent was provided by parents. Interviews with participants under 18 were conducted by telephone for comfort and privacy, as approved by the ethics committee. A parent was present but instructed not to answer for the child; they could clarify questions if needed, while responses came directly from the adolescent to capture their own perspective.

### Sampling strategy

This study used a two-stage stratified random sampling method to ensure a representative sample of older adults couples across Juybar city. The city, with a population of approximately 34,000 and 4,050 older adults’ people, is served by three government health centers with complete household health records. In the first stage, stratified sampling was conducted using all three comprehensive urban health service centers as strata to ensure geographical coverage. In the second stage, simple random sampling was applied within each stratum. Eligible older adults’ couples were selected from the health records using a computerized random number generator in STATA version 16 (https://www.stata.com) to minimize selection bias.

### Sample size calculation

Based on the study objectives, sample size calculations were performed for all aims, and the largest required sample size was selected. Drawing from previous research^16^ and literature review regarding the strength of association between loneliness, social isolation, and family communication networks, the smallest observed correlation coefficient of 0.21 between loneliness and communication networks was used as the basis (choosing the minimal effect size to ensure maximum sample size). Considering a 95% confidence level and 80% statistical power, and applying a two-tailed hypothesis using G*Power software version 3 (https://www.psychologie.hhu.de/arbeitsgruppen/allgemeine-psychologie-und- arbeitspsychologie/gpower), the required sample size was calculated as 173 older adults. Accounting for a 10% attrition rate, a total of 190 participants were recruited. Ultimately, after excluding five individuals, data from 185 older adults and their families were analyzed. In addition to the older adults included in the first generation, the study also recruited 185 adult children (second generation) and 185 grandchildren (third generation), resulting in a total of 555 participants representing three generational levels within families.

### Data collection

**Fig. 1:**
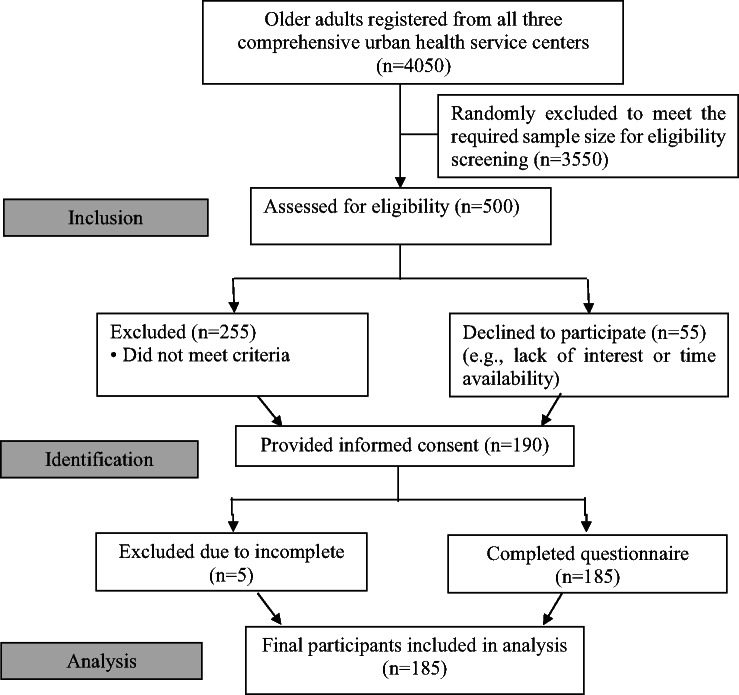
The study participant flow chart in line with the STROBE.

Following the issuance of an official letter of introduction by the Vice-Chancellors for Research at Babol and Mazandaran Universities of Medical Sciences, the researcher was formally introduced to all three urban comprehensive health service centers in Jouibar, which together represented the full set of such facilities in the city. In collaboration with healthcare providers at each center, a list of eligible older adults was compiled from health records. A stratified random sampling method was employed, with each center considered a separate stratum. Within each stratum, and proportional to its population size, individuals were selected by simple random sampling using a computerized random-number generator in STATA. At the time of selection, a randomized reserve list of additional eligible individuals from the same stratum was also generated. During the first telephone contact, inclusion and exclusion criteria were applied, including functional status. If a selected person was unavailable, ineligible, or refused participation after repeated contact attempts, the first available individual on the reserve list was contacted as a replacement. Potentially eligible participants who met these criteria were then informed about the study objectives and invited to attend the health center. At the center, after addressing participants’ questions, written informed consent was obtained, and the first-generation questionnaires were administered. Participants could either complete them independently in the presence of the researcher or respond through an interview. For the second and third generations, questionnaires were completed on-site if children were present, otherwise through telephone interviews. Figure 1 shows the flow chart of the participant study.

### Survey questionnaires

Data were collected via face-to-face interviews using structured questionnaires. The following validated instruments were administered:

### Sociodemographic and health status across generations

The study included three generations: older adults (G1), their adult children (G2), and grandchildren (G3). For G1 participant, data were collected on age, gender, counts of children, daughters, sons, grandchildren, granddaughters, and grandsons, education, occupation status, pension receipt, living arrangements, residence place (urban vs. rural), smoking. Financial sufficiency was assessed by asking participants to rate their household income’s adequacy for basic expenses, with responses categorized as sufficient or insufficient. Exercise habits were also determined based on whether participants met the WHO recommendation of ≥ 150 min of moderate-intensity aerobic activity weekly. Other variables included chronic diseases, multimorbidities (≥ two chronic diseases), polypharmacy (≥ five medications daily), self-rated health compared to peers, hospitalizations in the past year. The G2 characteristics included age, gender, education, occupation status, residence place, child–parent distance, perception of parents’ health, smoking, financial sufficiency, exercise, underlying diseases, multimorbidities, polypharmacy, self-rated health, marriage duration, and number of children, daughters, and sons, along with prevalent chronic diseases. Proximity was operationalized as adult children living in the same neighborhood versus residing in another neighborhood, city, or province, reflecting the importance of neighborhood-level closeness in Iranian society. For the G3, characteristics recorded were age, gender, education, and occupation status. In this study, the term multigenerational family refers to three consecutive generations, older adults, their adult children, and grandchildren, linked through active family ties rather than necessarily sharing a single household.

### Social isolation

The Social Isolation Questionnaire, developed by Chalabi and Amirkafi (2004), was employed to assess perceived social isolation in this study^17^. This culturally adapted instrument consists of 19 items, each rated on a five-point Likert scale ranging from 1 (strongly disagree) to 5 (strongly agree). The tool captures four core dimensions of social isolation: social loneliness (items 1–6), reflecting feelings of detachment from others; helplessness (items 7–10), indicating difficulties in establishing and maintaining relationships; social despair (items 11–15), representing pessimism about improving social ties; and reduced social tolerance (items 16–19), assessing difficulty in enduring social interactions. The total score ranges from 19 to 95, with higher scores reflecting greater perceived isolation. Scoring is reversed for items 1, 2, 4, 14, 15, 16, 17, 18, and 19. The scale has demonstrated satisfactory psychometric properties^18^. Following the method used in previous validation studies^18^, a total score of 57 or higher (corresponding to the midpoint of the scale’s range) was used to indicate social isolation. The same criterion was applied to the subscales.

### Loneliness scale

The De Jong Gierveld Loneliness Scale (1985) is a validated instrument designed to assess loneliness in two distinct dimensions: social and emotional^19^. The original version comprises 11 items, with 6 items focused on emotional loneliness and 5 items evaluating social loneliness. Responses are captured on a five-point Likert scale (ranging from 0 = never to 4 = always), with total scores ranging from 0 to 11, where higher scores reflect greater levels of loneliness. In a study conducted by Hosseini and colleagues (2021), the Persian version of this scale was translated and psychometrically evaluated among Iranian older adults. During the cultural adaptation process, three items were removed, resulting in a final version with 8 items: 5 items for social loneliness and 3 items for emotional loneliness. The total score ranges from 0 to 8, with higher scores indicating greater levels of loneliness. Both exploratory and confirmatory factor analyses supported the two-factor structure of the scale, and the results demonstrated satisfactory reliability^20^. Participants were categorized as “lonely” or “not lonely” using the scale’s midpoint, with scores ≥ 4 (range 0–8) indicating loneliness. This distribution-derived threshold represents a conventional epidemiological approach in the absence of validated cut- points.

### Activities of daily living

Katz ADL Index^21^ was used to confirm the participants’ functional independence. This test assesses a person’s ability to perform basic life activities in the six functions of bathing, dressing, toileting, transferring, continence, and feeding. Clients are scored yes/no for independence in each of the six functions. A score of 6 indicates full function, 4 indicates moderate impairment, and 2 or less indicates severe functional impairment^22^.

### Analysis

Data normality was initially assessed using the Kolmogorov-Smirnov test to determine the appropriate statistical approach. For comparing mean values between groups, the independent t-test was applied under the assumption of equal variances, while for ANOVA, the assumption of homogeneity of variances was tested using Levene’s test. The chi-square test was used to assess associations between categorical variables. Both univariate and multivariate linear regression analyses were employed to examine key variables, with all study factors considered. Variables showing *p* < 0.2 in univariate analysis were included in the multivariate models. Standardized and unstandardized regression coefficients, along with 95% confidence intervals, were reported. All analyses were conducted using SPSS version 26 (https://www.ibm.com/products/spss-statistics), with a significance level set at *p* < 0.05.

## Results

The mean age of the older adults’ participants was 67.2 ± 5.8 years, ranging from 60 to 90. On average, they reported having 4.7 ± 1.5 children (range: 2–10) and 2.3 ± 1.1 grandchildren (range: 0–5). Among the first-generation participants, 53% were female, 68% were unemployed, and 73% lived exclusively with their spouse. Approximately 45% received retirement pensions, and 62% reported having income equal to their expenses. Additionally, 31% engaged in regular physical activity, 75% reported at least one chronic condition, nearly half had multimorbidity, and 25% experienced polypharmacy. The most prevalent chronic illnesses were hypertension (49.7%), diabetes (35.7%), and cardiovascular diseases (29.7%) (Table 1). ). To assess the internal consistency of the loneliness and social isolation scales, omega was calculated, yielding values of 0.788 and 0.877, respectively, indicating acceptable internal consistency for each of these scales.

The mean age of second-generation participants was 40.8 ± 5.9 years (range: 28–56). Their average duration of marriage was 19.3 ± 6.0 years (range: 8–35), and they typically had one child. Among this group, 68% were women, 47% had completed high school or less, and 43% were homemakers. Around 60% lived in the same neighborhood as their older adults parent. In total, 20% reported having a chronic condition, and 7% had multimorbidity. The most common health issues were hypertension (9.2%) and gastrointestinal disorders (5.4%). The average age of the grandchildren was 15.4 ± 5.3 years (range: 7–31). Approximately 60% were female, and 80% were students (Tables 2 and 3).

The prevalence of loneliness among the older adults was 68.6%, while social isolation was reported in 29% of participants. The means, standard deviations, and frequency (%) of these variables across subgroups are presented in Supplementary Table 1.

In the multivariate regression model used to examine the relationships between loneliness, social isolation, and the variables in the study, all potential confounders were initially included, regardless of their statistical significance in the univariate models. However, upon further examination, it was noted that some key variables, such as gender and education, did not exhibit significant associations with the outcomes. Detailed results of these analyses are provided in Supplementary 2 and 3.

Given the limited sample size and the need to enhance the statistical power of the analysis, a second approach was also considered. In this scenario, only variables with a p-value less than 0.2 in the univariate analysis were included in the multivariate models. This strategy was employed to account for potentially weak but theoretically important confounders and to mitigate the risk of excluding variables that, despite not reaching conventional significance thresholds, may still have relevant associations with the outcomes. The results of this adjusted analysis are presented in Tables 4 and 5.

In multivariable analysis, financial insufficiency was significantly associated with increased loneliness. Older adults individuals whose income met or exceeded their expenses reported lower levels of loneliness compared to those with insufficient income (B = − 0.69, SE = 0.33, 95% CI: − 1.34 to − 0.035, *p* = 0.039). Regarding social isolation, multivariable analysis indicated that place of residence was the only significant predictor. Older adults residing in rural areas reported lower levels of social isolation compared to their urban counterparts (B = − 4.43, SE = 2.07, 95% CI: − 8.52 to − 0.35, *p* = 0.033) (Table 4).

Table 5 presents the association between second- and third-generation characteristics and the older adults’ loneliness and social isolation. In multivariable analysis, the only significant predictor among second-generation characteristics was geographic proximity to the older adults’ parent. Older adults whose children lived in a different neighborhood experienced higher levels of social isolation than those whose children lived nearby (B = 3.73, SE = 1.59, 95% CI: 0.56 to 6.87, *p* = 0.020), underscoring the importance of family proximity for social access. Although financial sufficiency of the adult child showed a potential association with parental social isolation (B = 2.92, *p* = 0.115), this was not statistically significant.

Other variables related to the adult children, including age, gender, education level, marital duration, number of children, employment status, financial sufficiency, multimorbidity, and polypharmacy, showed no statistically significant relationship with the older adults’ loneliness or social isolation. Similarly, none of the individual characteristics of the grandchildren, age, gender, educational status, or occupation, were significantly related to the grandparents’ loneliness or social isolation (all *p* > 0.05). While univariable analysis indicated that higher educational levels among grandchildren were associated with greater loneliness scores in grandparents (B = 0.53, *p* = 0.117), this association did not remain significant in the multivariable model (*p* = 0.751).

**Table 1:** Demographic and clinical features of the older adult (*n* = 185).

Variable		Mean ± SD Or *N* (%)	Loneliness Mean ± SD	Social Isolation Mean ± SD
Age (year)	60–74	165 (89.2)	4.9 ± 2.1	44.8 ± 10.5
	≥ 75	20 (10.8)	4.3 ± 1.5	42.4 ± 12.8
Gender	Male	87 (47)	4.8 ± 1.9	44.4 ± 11.7
	Female	98 (53)	4.9 ± 2.1	44.6 ± 10.0
Education level	Illiterate	69 (37)	5.1 ± 1.9	44.7 ± 11.7
	Diploma or less	105 (56.8)	4.7 ± 2.1	44.3 ± 10.4
	College	11 (5.9)	5.2 ± 2.1	45.8 ± 8.8
Residence place	Urban	158 (85.4)	4.8 ± 2.1	44.9 ± 10.6
	Rural	27 (14.6)	5.0 ± 1.6	41.9 ± 11.2
Occupation status	Employed	58 (31.4)	4.9 ± 2.1	44.0 ± 12.5
	Unemployed	127 (68.6)	4.8 ± 2.0	44.8 ± 9.9
Pension	Yes	84 (45.4)	4.9 ± 2.0	44.9 ± 10.7
	No	101 (54.6)	4.9 ± 2.0	44.3 ± 10.9
Living arrangement	With spouse	135 (73)	4.8 ± 2.1	44.3 ± 10.6
	With spouse and children	50 (27)	5.1 ± 2.0	45.2 ± 11.4
Smoking status	Current smoker	16 (8.6)	5.0 ± 2.0	46.7 ± 11.8
	Former smoker	10 (5.4)	4.9 ± 1.7	44.0 ± 10.1
	Non-smoker	159 (85.9)	4.8 ± 2.1	44.4 ± 10.8
Financial sufficiency	Adequate	133 (71.9)	4.7 ± 2.1*	44.1 ± 10.8
	Inadequate	52 (28.1)	5.4 ± 1.9	45.6 ± 10.9
Exercise	Yes	59 (31.9)	5.0 ± 2.0	45.5 ± 11.1
	No	126 (68.1)	4.8 ± 2.1	44.1 ± 10.6
Underlying disease	Yes	139 (75.1)	5.0 ± 2.0	44.4 ± 10.9
	No	46 (24.9)	4.4 ± 2.1	45.1 ± 10.5
Multimorbidities	Yes	94 (50.8)	5.0 ± 1.9	45.8 ± 11.5
	No	91 (49.2)	4.7 ± 2.1	43.3 ± 9.8
Polypharmacy	Yes	46 (24.9)	5.1 ± 2.0	44.7 ± 11.7
	No	139 (75.1)	4.8 ± 2.0	44.5 ± 10.5
Self-rated health compared to peers	Better	53 (28.6)	4.6 ± 2.1	42.9 ± 9.8
	Same	87 (47)	4.9 ± 2.0	44.1 ± 11.0
	Worse	45 (24.3)	5.1 ± 2.1	47.4 ± 11.1
Hospitalization (past year)	Yes	35 (18.9)	5.1 ± 2.0	44.8 ± 11.8
	No	150 (81.1)	4.8 ± 2.0	44.5 ± 10.6
Children count	≤ 3	46 (24.9)	5.0 ± 1.9	45.5 ± 8.9
	> 4	139 (75.1)	4.8 ± 2.1	44.2 ± 11.3
Sons count	Range: 0–7	2.4 ± 1.3		
Daughters count	Range: 0–5	2.9 ± 1.1		
Grandchildren count	Range: 1–23	6.83 ± 3.7		
Granddaughters Count	Range: 0–11	3.5 ± 2.4		
Grandsons count	Range: 0–12	3.4 ± 2.5		

Polypharmacy: the regular use of five or more medications concurrently. Multimorbidity: the co-existence of two or more chronic conditions in an individual, without one condition designated as primary.

X^2^, T-test, Anova.

*p < 0.05.

**Table 2:** Demographic and clinical features of the adult children and their association with social isolation and loneliness among their elderly parents (*n* = 185).

Variable		Mean ± SD Or *N* (%)	Loneliness Mean ± SD	Social Isolation Mean ± SD
Age (years)	28–40	102 (55.1)	4.6 ± 2.1	43.1 ± 10.3*
	41–56	83 (44.9	5.2 ± 1.9	46.3 ± 11.2
Gender	Male	127 (68.8)	5.1 ± 1.9	45.4 ± 11.0
	Female	58 (31.4)	4.7 ± 2.1	44.1 ± 10.7
Education level	Illiterate	2 (1.1)	5.5 ± 2.1	38.5 ± 2.1
	Diploma or less	85 (56.2)	4.9 ± 1.9	44.9 ± 10.8
	College	79 (42.7)	4.8 ± 2.1	44.4 ± 10.9
Occupation status	Employed	104 (56.2)	4.9 ± 2.0	44.4 ± 10.9
	Unemployed	81 (43.8)	4.8 ± 2.1	44.7 ± 10.6
Living arrangement	With spouse	11 (5.9)	5.7 ± 2.3	42.4 ± 8.1
	With spouse and children	174 (94.1)	4.8 ± 2.0	44.7 ± 10.9
Residence place	Urban	171 (92.4)	4.9 ± 2.0	44.5 ± 10.8
	Rural	14 (7.6)	4.6 ± 2.1	44.9 ± 11.0
Child–parent distance	Same neighborhood	111 (60)	4.8 ± 1.9	46.7 ± 12.0*
	Other*	74 (40)	5.0 ± 2.3	43.7 ± 9.6
Child’s perception of parents’ health	Poor	9 (4.9)	4.9 ± 2.0	44.5 ± 10.8
	Moderate	107 (57.8)	5.0 ± 2.0	44.0 ± 11.4
	Good	69 (37.3)	4.7 ± 2.0	45.1 ± 9.4
Smoking status	Yes	8 (4.3)	4.9 ± 2.0*	44.9 ± 10.8*
	No	177 (95.7)	3.9 ± 1.7	36.5 ± 9.4
Financial sufficiency	Adequate	138 (74.6)	4.8 ± 2.0	45.4 ± 10.8
	Inadequate	47 (25.4)	5.1 ± 2.2	42.2 ± 10.5
Exercise	Yes	69 (37.3)	4.8 ± 2.1	45.0 ± 10.8
	No	116 (62.7)	4.9 ± 2.0	44.3 ± 10.8
Underlying disease	Yes	38 (20.5)	5.1 ± 2.2	45.9 ± 11.7
	No	147 (79.5)	4.8 ± 2.0	44.2 ± 10.5
Multimorbidities	Yes	13 (7)	5.1 ± 2.4	46.8 ± 12.1
	No	172 (93)	4.8 ± 2.0	44.4 ± 10.7
Self-rated health compared to peers	Better	40 (21.6)	4.9 ± 2.2	42.7 ± 10.2
	Same	132 (71.4)	4.8 ± 2.0	44.0 ± 10.8
	Worse	13 (7)	5.4 ± 2.1	45.8 ± 12.5
Self-perceived spirituality	Low	14 (7.6)	5.0 ± 2.5	50.3 ± 9.1*
	Moderate	97 (52.4)	4.9 ± 1.9	45.2 ± 10.4
	High	74 (40)	4.8 ± 2.2	44.5 ± 10.8
Marriage duration (year)	8–15	64 (34.6)	4.7 ± 2.3	43.8 ± 10.8
	15–25	86 (46.5)	4.9 ± 1.9	44.5 ± 11.3
	25–35	35 (18.9)	5.0 ± 1.9	45.2 ± 10.6
Children count	Range: 1–5	1.9 ± 0.62		
Daughters count	Range: 0–3	0.98 ± 0.66		
Sons count	Range: 0–2	0.90 ± 0.68		

*Same or different city/province.

X^2^, T-test, Anova.

*p < 0.05.

**Table 3:** Demographic characteristics of the grandchildren (*n* = 185).

Variable		Mean ± SD Or *N* (%)	Loneliness Mean ± SD	Social isolation Mean ± SD
Age (years)	7–19	145 (78.4)	4.8 ± 2.1	44.4 ± 10.9
	20–31	40 (21.6)	5.2 ± 1.9	45.1 ± 10.4
Gender	Male	110 (59.5)	4.7 ± 2.0	43.6 ± 11.1
	Female	75 (40.5)	5.0 ± 2.0	45.2 ± 10.5
Education level	Primary	61 (33)	4.5 ± 2.3	61 ± 42.3
	Secondary/higher	75 (40.6)	4.9 ± 1.9	75 ± 45.2
	College	49 (26.5)	5.2 ± 1.9	49 ± 46.4
Occupation status	Student	133 (71.9)	4.7 ± 2.1	44.0 ± 10.9
	University student	34 (18.4)	5.4 ± 1.8	46.9 ± 10.3
	Other	18 (9.8)	4.9 ± 2.0	44.3 ± 10.7

X^2^, T-test, Anova.

*p < 0.05.

**Table 4:** The association between older adults’ characteristics, loneliness, and social isolation in univariate and multivariate analyses.

Variable name	Loneliness						Social isolation					
	Univariate analysis (crude effects)			Multivariate analysis (adjusted effects)			Univariate analysis (crude effects)			Multivariate analysis (adjusted effects)		
	B (SE)	95% CI	p	B (SE)	95% CI	p	B (SE)	95% CI	p	B (SE)	95% CI	p
Age	− 0.02 (0.02)	− 0.07 to 0.03	0.441	–			− 0.08 (0.13)	− 0.35 to 0.18	0.536	–		
Gender (female/male)	− 0.09 (0.30)	− 0.68 to 0.49	0.756	–			− 0.21 (1.59)	− 3.35 to 2.92	0.891	–		
Education level (illiterate/literate)	− 0.41 (0.30)	− 1.02 to 0.19	0.177	− 0.38 (0.31)	− 0.98 to 0.23	0.221	− 0.25 (1.64)	–	0.880			
Children count	0.09 (0.09)	− 0.09 to 0.29	0.322	–			0.33 (0.52)	− 0.69 to 1.35	0.523	–		
Grandchildren count	− 0.02 (0.04)	− 0.10 to 0.06	0.603	–			− 0.08 (0.21)	− 0.50 to 0.33	0.689	–		
Living place (rural/urban)	0.17 (0.42)	− 0.66 to 1.00	0.688	–			− 3.01 (2.23)	− 7.43 to 1.39	0.179	− 4.43 (2.07)	− 8.52 to − 0.35	0.033
Occupation status (unemployed/employed)	− 0.08 (0.32)	− 0.55 to 0.72	0.788	–			− 0.73 (1.71)	− 4.11 to 2.63	0.667	–		
Living arrangement (with spouse/with spouse and child)	0.33 (0.33)	− 0.32 to 1.00	0.318	–			0.84 (1.78)	− 2.67 to 4.37	0.635	–		
Financial sufficiency (income < expenses/income ≥ expenses)	− 0.71 (0.32)	− 1.36 to − 0.06	0.032	− 0.69 (0.33)	− 1.34 to − 0.035	0.039	− 1.41 (1.76)	− 4.90 t0 2.07	0.423			
Multimorbidities (no/yes)	0.34 (0.29)	− 0.25 to 0.93	0.207	–			2.47 (1.57)	− 0.64 to 5.58	0.119	0.57 (1.46)	− 2.32 to 3.46	0.698
Polypharmacy (no/yes)	0.31 (0.34)	− 0.37 to 0.99	0.372	–			0.17 (1.83)	− 3.44 to 3.80	0.923	–		

*The results of the multivariate analysis are adjusted for variables with a p-value of less than 0.2 in the univariate analysis.

**Table 5:** Association of loneliness and social isolation with second & third-generation characteristics in univariate and multivariate analysis

Variable name	Loneliness						Social isolation					
	Univariate analysis (crude effects)			Multivariate analysis (adjusted effects)			Univariate analysis (crude effects)			Multivariate analysis (adjusted effects)		
	B (SE)	95% CI	*p*	B (SE)	95% CI	*p*	B (SE)	95% CI	*p*	B (SE)	95% CI	*p*
Second generation												
Age	0.04 (0.02)	− 0.005 to 0.09	0.080	0.03 (0.02)	− 0.01 to 0.08	0.133	0.20 (0.13)	− 0.05 to 0.47	0.125	0.15 (0.14)	− 0.12 to 0.42	0.265
Gender (female/male)	0.36 (0.32)	− 0.27 to 0.99	0.261	–			1.29 (1.70)	− 2.07 to 4.67	0.449	–		
Education level (up to diploma/university)	− 0.08 (0.30)	− 0.67 to 0.51	0.790	–			− 0.39 (1.59)	− 3.52 to 2.74	0.807	–		
Marriage duration	0.012 (0.02)	− 0.03 to 0.06	0.625	–			0.10 (0.13)	− 0.16 to 0.36	0.446	–		
Children count	0.05 (0.23)	− 0.41 to 0.52	0.821	–			− 0.73 (1.26)	− 3.23 to 1.76	0.560	–		
Occupation status (unemployed/employed)	0.04 (0.30)	− 0.54 to 0.64	0.874	–			− 0.35 (1.60)	− 3.51 to 2.80	0.824	–		
Financial sufficiency (income < expenses/income ≥ expenses)	− 0.36 (0.34)	− 1.04 to 0.31	0.287	–			3.17 (1.80)	− 0.39 to 6.74	0.081	2.92 (1.84)	− 0.72 to 6.55	0.115
Distance from parents (same neighborhood/other)	0.26 (0.30)	− 0.33 to 0.86	0.385	–			3.60 (1.59)	0.44 to 6.75	0.025	3.73 (1.59)	0.559 to 6.87	0.020
Multiomorbidities (no/yes)	0.24 (0.58)	− 0.91 to 1.39	0.683	–			2.39 (3.10)	− 3.72 to 8.51	0.441	–		
Polypharmacy (no/yes)	0.28 (0.33)	− 0.37 to 0.94	0.400	–			0.09 (1.77)	− 3.41 to 3.60	0.958	–		
Third generation												
Age	0.04 (0.02)	− 0.008 to 0.10	0.092	0.034 (0.05)	− 0.05 to 0.12	0.449	0.19 (0.14)	− 0.09 to 0.49	0.184	0.10 (0.24)	− 0.37 to 0.57	0.673
Gender (female/male)	− 0.31 (0.30)	− 0.91 to 0.28	0.302	–			− 1.55 (1.61)	− 4.74 to 1.62	0.335	–		
Education level (up to diploma/university)	0.53 (0.23)	− 0.13 to 1.19	0.117	0.17 (0.54)	− 0.89 to 1.23	0.751	0.172	− 1.07 to 5.99	0.172	1.52 (2.86)	− 4.13 to 7.16	0.597
Occupation (student/other)	0.10 (0.50)	− 0.89 to 1.09	0.843	–			− 0.23 (2.68)	− 5.51 to 5.05	0.932	–		

*The results of the multivariate analysis are adjusted for variables with a p-value of less than 0.2 in the univariate analysis.

## Discussion

The present study examined the interconnection between both individual and multigenerational family characteristics and older adults’ experiences of loneliness and social isolation. Whereas previous research has largely focused on psychosocial or health-related risk factors, our multigenerational approach provides an advantage in terms of understanding family-related factors in late-life well-being. The findings reveal that financial security and geographical location of the first generation (older adults), as well as the geographical closeness of adult children in the second generation, were most strongly associated with loneliness and social isolation, respectively. Contrary to some earlier presumptions, the personal characteristics of grandchildren did not have a considerable influence on these outcomes.

The significant association between financial adequacy and reduced perceptions of loneliness is consistent with evidence from both global and local contexts. Economic security not only removes the stress of basic needs but also promotes social engagement by enabling mobility, access to basic services, and participation in communal activities. Earlier studies in Spain and Brazil, for instance, found that older adults with adequate income reported lower levels of loneliness as well as greater perceived autonomy^23,24^. Iranian data also highlight financial challenges as a major cause of elder distress^25^. Our results are consistent with these observations, suggesting that income security is associated with reduced loneliness and may serve a protective function, even in the context of rich familial cultures like Iran’s.

The result that rural residency was linked to less social isolation might be unexpected, based on expectations of scarce resources in rural communities. In much of Iran’s rural environment, though, close-knit social fabrics and stable local networks afford regular, informal contact. This is consistent with findings in China and Canada, where older persons living in rural areas had stronger neighborhood bonds and less social withdrawal than urban residents^26,27^. Urban older persons, in contrast, are more likely to encounter fragmented living conditions and undermined intergenerational support^28^. This trend may also be a reflection of the breakdown in traditional support systems in urban Iran under the influence of modernization and migration^14^.

One of the most innovative aspects of our study is the multigenerational analysis, in which geographic closeness of adult children was strongly associated with less social isolation in their aging parents. This concurs with previous evidence from East Asian and European research, in which co-residence or close proximity with adult children was linked to more frequent contact and superior psychological outcomes in older persons^29,30^. Plausibly, near physical distance allows for both instrumental and emotional support, ranging from daily chores to impromptu visits, each of which may serve as buffers against social isolation. A recent Korean study validated that older adults who received face-to-face contact at least three times weekly from proximal children had significantly higher social connectedness scores^31^. Although long-distance communication is possible with modern technology, it seems that the physical presence of children continues to hold a special emotional and instrumental worth. In Iranian cultural context, proximity also reflects respect and filial duty, which can be psychologically rewarding to aging parents.

Conversely, there were no notable associations between grandchildren’s characteristics and their grandparents’ social outcomes. While younger generations might be assumed to offer emotional companionship, especially in collectivist cultures, their net effect might hinge on interaction quality and frequency instead of demographic characteristics such as age, gender, or education. Further, most grandchildren, particularly those in adolescence or young adulthood, might lack time or the emotional space to meaningfully connect with older relatives. This portends the necessity for research to move beyond static variables and examine dynamic, qualitative elements of intergenerational relationships like affection, activity sharing, or affective reciprocity.

Similarly, most of the characteristics of adult children, educational attainment, income, health status, and occupational status, were not significantly associate with either loneliness or social isolation in the older adults. Although this result might seem surprising, it highlights once again the idea that structural aspects of family interaction (e.g., geographic distance) are often more important than individual traits. In addition, the absence of emotional intimacy measures in this study may help explain the lack of some expected correlations. It is possible, for example, that distant children who maintain an affectionate emotional relationship may be perceived as more comforting than nearby children who interact infrequently.

The novelty of our study lies in its tri-generational design, enabling a more nuanced understanding of family systems and their diverse influences on older adults’ social health. In contrast to most studies that view older adults as isolated individuals, our model acknowledges their embeddedness in active intergenerational networks, a perspective that is especially relevant in collectivist cultures. In addition, this approach aims to address current gaps in Iranian research, where previous studies have often assessed loneliness without incorporating multigenerational data^32,33^.

The results have practical applications. Interventions to decrease loneliness and isolation should address not only individual psychological characteristics but also strive to enhance family networks. Urban planners might consider incentivizing multigenerational housing or subsidizing housing for adult children who want to live close to their older adults parents. Community programs could provide structured intergenerational interaction opportunities, such as storytelling workshops, shared gardening, or digital training for elders to more easily connect with far-away family. In rural communities, maintaining community cohesion through neighborhood centers or mobile health teams might sustain the protective effects already inherent in these environments. This study is limited by its cross-sectional design, which precludes causal inference, and by the reliance on self-reported data that may be influenced by recall or social desirability bias. The focus on married older adults with married children and grandchildren, while ensuring comparability for tri-generational network analysis, reduces generalizability; the experiences of widowed, divorced, or never-married older adults may differ, particularly regarding loneliness and social isolation. Despite these limitations, the large sample size, rigorous sampling strategy, and unique multigenerational scope strengthen the validity and relevance of the findings.

## Conclusion

This study reveals a critical distinction: while financial adequacy is a pivotal buffer against loneliness in older adults, residing in rural areas and having adult children living nearby are specific protective factors against social isolation. The lack of significant associations with a wide range of other second- and third-generation characteristics suggests that the intergenerational dynamics of elder well-being are not broadly mediated by the socio-economic or health status of younger generations, but rather by concrete, actionable factors like economics and geographic proximity. These findings advocate for targeted policy and intervention strategies that prioritize economic support for the older adults and community planning that facilitates family co-residence or proximity, moving beyond broader but less impactful multigenerational approaches.

## Supplementary Information

Below is the link to the electronic supplementary material.

Supplementary Material 1

## Data Availability

The datasets used and/or analyzed during the current study are available from the corresponding author upon reasonable request.
