# Supplementary material for: The association of multigenerational family characteristics with loneliness and social isolation in older adults
Source: Sci Rep. 2025 Dec 4;16:735. doi: 10.1038/s41598-025-30227-7 (PMC12780082; doi:10.1038/s41598-025-30227-7)
Supplement: Supplementary file 1 — Supplementary Material 1 [file 41598_2025_30227_MOESM1_ESM.docx]

**Supplementary Table 1.** Frequency and mean of social isolation and loneliness dimensions among the older adults

| **Variable** | **Minimum-**  **Maximum** | **Mean ± SD** | **Frequency**  **(percentage)** |
| --- | --- | --- | --- |
| **Loneliness** | | | |
| Emotional | 0-3 | 2.3 ± 0.93 | 150 (81.1) |
| Social | 0-5 | 2.5 ± 1.8 | 101 (54.6) |
| Total | 0-8 | 4.9 ± 2.0 | 127 (68.6) |
| **Social Isolation** | | | |
| Social Loneliness | 6-24 | 13.5 ± 3.7 | 30 (16.2) |
| Helplessness | 4-16 | 10.2 ± 2.7 | 61 (33) |
| Social Despair | 5-21 | 11.3 ± 3.3 | 34 (18.4) |
| Reduced Social Tolerance | 4-17 | 9.6 ± 3.2 | 50 (27) |
| Total | 25-72 | 44.5 ± 10.8 | 29 (15.7)) |

**Supplementary Table 2.** The association between older adults’ characteristics, loneliness, and social isolation in univariate and multivariate analyses

| **Variable Name** | **Loneliness** | | | | | | | **Social Isolation** | | | | | | | |
| --- | --- | --- | --- | --- | --- | --- | --- | --- | --- | --- | --- | --- | --- | --- | --- |
|  | **Univariate Analysis**  **(Crude Effects)** | | | **Multivariate Analysis (Adjusted Effects)** | | | | **Univariate Analysis**  **(Crude Effects)** | | | **Multivariate Analysis (Adjusted Effects)** | | | | |
|  | **B (SE)** | **95% CI** | **p** | **B (SE)** | **95% CI** | | **p** | **B (SE)** | **95% CI** | **p** | **B (SE)** | **95% CI** | | **p** |  |
| Age | -0.02 (0.02) | -0.07 to 0.03 | 0.441 | -0.03 (0.03) | -1.00 to 0.03 | | 0.289 | -0.08 (0.13) | -0.35 to 0.18 | 0.536 | -0.09 (0.17) | | -0.44 to 0.25 | 0.589 |  |
| Gender  (Female/Male) | -0.09 (0.30) | -0.68 to 0.49 | 0.756 | -0.006 (0.40) | | -0.80 to 0.79 | 0.989 | -0.21 (1.59) | -3.35 to 2.92 | 0.891 | 0.20 (2.17) | | -4.08 to 4.50 | 0.924 |  |
| Education Level  (Illiterate/Literate) | -0.41 (0.30) | -1.02 to 0.19 | 0.177 | -0.45 (0.36) | -1.16 to 0.25 | | 0.206 | -0.25 (1.64) | -3.49 to  2.99 | 0.880 | 0.09 (1.93) | | -3.73 to 3.91 | 0.962 |  |
| Children count | 0.09 (0.09) | -0.09 to 0.29 | 0.322 | 0.24 (0.14) | | -0.37 to 0.53 | 0.088 | 0.33 (0.52) | -0.69 to 1.35 | 0.523 | 0.98 (0.77) | | -0.55 to 2.51 | 0.208 |  |
| Grandchildren Count | -0.02 (0.04) | -0.10 to 0.06 | 0.603 | -0.07 (0.06) | | -0.20 to 0.04 | 0.230 | -0.08 (0.21) | - 0.50 to 0.33 | 0.689 | -0.30 (0.33) | | -0.97 to 0.36 | 0.368 |  |
| Living Place  (Rural/Urban) | 0.17  (0.42) | -0.66 to  1.00 | 0.688 | 0.27 (0.44) | | -0.60 to 1.14 | 0.545 | -3.01 (2.23) | -7.43 to 1.39 | 0.179 | -2.65 (2.39) | | -7.38 to 2.08 | 0.270 |  |
| Occupation Status (Unemployed/Employed) | -0.08 (0.32) | -0.55 to 0.72 | 0.788 | 0.02 (0.42) | | -0.56 to 1.12 | 0.514 | -0.73 (1.71) | -4.11 to 2.63 | 0.667 | -0.02 (2.29) | | -4.56 to 4.50 | 0.990 |  |
| Living Arrangement  (With Spouse / With Spouse and Child) | 0.33 (0.33) | -0.32 to 1.00 | 0.318 | 0.10 (0.36) | | -0.60 to 0.82 | 0.767 | 0.84 (1.78) | -2.67 to 4.37 | 0.635 | -0.36 (1.95) | | -4.22 to 3.50 | 0.854 |  |
| Financial Sufficiency  (Income < Expenses / Income ≥ Expenses) | -0.71 (0.32) | -1.36 to -0.06 | 0.032 | -0.67 (0.34) | -1.35 to 0.01 | | 0.056 | -1.41 (1.76) | -4.90 t0 2.07 | 0.423 | -1.31 (1.87) | | -5.02 to 2.38 | 0.484 |  |
| Multimorbidities  (No/Yes) | 0.34 (0.29) | -0.25 to 0.93 | 0.207 | 0.21 (0.36) | | -0.50 to 0.92 | 0.558 | 2.47 (1.57) | -0.64 to 5.58 | 0.119 | 2.81 (1.94) | -1.02 to 6.66 | | 0.150 |  |
| Polypharmacy  (No/Yes) | 0.31 (0.34) | -0.37 to 0.99 | 0.372 | 0.16 (0.41) | | -0.65 to 0.98 | 0.686 | 0.17 (1.83) | -3.44 to 3.80 | 0.923 | -1.73 (2.23) | | -6.13 to 2.67 | 0.439 |  |

* The results of the multivariate analysis are adjusted for all variables in the univariate analysis.

**Supplementary Table 3.** Association of loneliness and social isolation with second & third-generation characteristics in univariate and multivariate analysis

| **Variable Name** | **Loneliness** | | | | | | **Social Isolation** | | | | | |
| --- | --- | --- | --- | --- | --- | --- | --- | --- | --- | --- | --- | --- |
|  | **Univariate Analysis**  **(Crude Effects)** | | | **Multivariate Analysis (Adjusted Effects)** | | | **Univariate Analysis**  **(Crude Effects)** | | | **Multivariate Analysis (Adjusted Effects)** | | |
|  | **B**  **(SE)** | **95% CI** | **p** | **B (SE)** | **95% CI** | **p** | **B (SE)** | **95% CI** | **p** | **B (SE)** | **95% CI** | **p** |
| **Second Generation** | | | | | | | | | | | | |
| Age | 0.04 (0.02) | -0.005 to 0.09 | 0.080 | 0.09 (0.40) | 008 to 0.18 | 0.032 | 0.20 (0.13) | -0.05 to 0.47 | 0.125 | 0.18 (0.23) | -0.28 to 0.65 | 0.443 |
| Gender  (Female/Male) | 0.36 (0.32) | -0.27 to 0.99 | 0.261 | 0.29 (0.42) | -0.54 to 1.14 | 0.486 | 1.29 (1.70) | -2.07 to 4.67 | 0.449 | 2.62 (2.24) | -1.80 to 7.06 | 0.244 |
| Education Level  (Up to Diploma / University) | -0.08 (0.30) | -0.67 to 0.51 | 0.790 | -0.14 (0.34) | -0.83 to 0.54 | 0.675 | -0.39 (1.59) | -3.52 to 2.74 | 0.807 | 0.01 (1.82) | -3.58 to 3.62 | 0.992 |
| Marriage Duration | 0.012 (0.02) | -0.03 to 0.06 | 0.625 | -0.05 (0.04) | -0.14 to 0.03 | 0.208 | 0.10 (0.13) | -0.16 to 0.36 | 0.446 | -0.01 (0.24) | -0.48 to 0.46 | 0.965 |
| Children count | 0.05 (0.23) | -0.41 to 0.52 | 0.821 | -0.03 (0.26) | -0.54 to 0.48 | 0.909 | -0.73 (1.26) | -3.23 to 1.76 | 0.560 | -1.15 (1.36) | -3.85 to 1.55 | 0.402 |
| Occupation Status (Unemployed/Employed) | 0.04 (0.30) | -0.54 to 0.64 | 0.874 | -0.34 (0.40) | -1.15 to 0.46 | 0.400 | -0.35 (1.60) | -3.51 to 2.80 | 0.824 | -2.13 (2.14) | -6.36 to 2.09 | 0.320 |
| Financial Sufficiency  (Income < Expenses / Income ≥ Expenses) | -0.36 (0.34) | -1.04 to 0.31 | 0.287 | -0.54 (0.36) | -1.27 to 0.18 | 0.140 | 3.17 (1.80) | -0.39 to 6.74 | 0.081 | 2.75 (1.92) | -1.05 to 6.55 | 0.156 |
| Distance from Parents (Same Neighborhood / Other) | 0.26 (0.30) | -0.33 to 0.86 | 0.385 | 0.20 (0.32) | -0.43 to 0.83 | 0.528 | 3.60 (1.59) | 0.44 to 6.75 | 0.025 | 3.86 (1.68) | 0.545 to 7.18 | 0.023 |
| Multiomorbidities  (No/Yes) | 0.24 (0.58) | -0.91 to 1.39 | 0.683 | 0.13 (0.69) | -1.22 to 1.50 | 0.842 | 2.39 (3.10) | -3.72 to 8.51 | 0.441 | 2.01 (3.63) | -5.15 to 9.18 | 0.580 |
| Polypharmacy  (No/Yes) | 0.28 (0.33) | -0.37 to 0.94 | 0.400 | 0.09 (0.38) | -0.66 to 0.86 | 0.801 | 0.09 (1.77) | -3.41 to 3.60 | 0.958 | -0.41 (2.03) | -4.42 to 3.59 | 0.839 |
| **Third Generation** | | | | | | | | | | | | |
| Age | 0.04 (0.02) | -0.008 to 0.10 | 0.092 | 0.05 (0.04) | -0.04 to 0.15 | 0.278 | 0.19 (0.14) | -0.09 to 0.49 | 0.184 | 021 (0.26) | -0.29 to 0.73 | 0.405 |
| Gender  (Female/Male) | -0.31 (0.30) | -0.91 to 0.28 | 0.302 | -0.30 (0.30) | -0.91 to 0.30 | 0.329 | -1.55 (1.61) | -4.74 to 1.62 | 0.335 | -1.55 (1.64) | -4.78 to 1.68 | 0.346 |
| Education Level  (Up to Diploma / University) | 0.53 (0.23) | -0.13 to 1.19 | 0.117 | 0.16 (0.54) | -0.90 to 1.23 | 0.759 | 0.172 | -1.07 to 5.99 | 0.172 | 1.23 (2.87) | -4.44 to 6.90 | 0.669 |
| Occupation  (Student / Other) | 0.10 (0.50) | -0.89 to 1.09 | 0.843 | -0.61 (0.61) | -1.82 to 0.59 | 0.314 | -0.23 (2.68) | -5.51 to 5.05 | 0.932 | -3.58 (3.25) | -9.99 to 2.82 | 0.271 |

* The results of the multivariate analysis are adjusted for all variables in the univariate analysis.
